# A high-flow nasal cannula system with relatively low flow effectively washes out CO_2_ from the anatomical dead space in a sophisticated respiratory model made by a 3D printer

**DOI:** 10.1186/s40635-018-0172-7

**Published:** 2018-03-15

**Authors:** Yu Onodera, Ryo Akimoto, Hiroto Suzuki, Masayuki Okada, Masaki Nakane, Kaneyuki Kawamae

**Affiliations:** Department of Anesthesiology, Faculty of Medicine, Yamagata University, 2-2-2 Iidanishi, Yamagata City, Yamagata Prefecture 990-9585 Japan

**Keywords:** High-flow nasal cannula, Washout effect, Rebreathing, Ventilation, Work of breathing, PEEP

## Abstract

**Background:**

Although clinical studies of the high-flow nasal cannula (HFNC) and its effect on positive end-expiratory pressure (PEEP) have been done, the washout effect has not been well evaluated. Therefore, we made an experimental respiratory model to evaluate the respiratory physiological effect of HFNC.

**Methods:**

An airway model was made by a 3D printer using the craniocervical 3D-CT data of a healthy 32-year-old male. CO_2_ was infused into four respiratory lung models (normal-lung, open- and closed-mouth models; restrictive- and obstructive-lung, open-mouth models) to maintain the partial pressure of end-tidal CO_2_ (P_ET_CO_2_) at 40 mmHg. HFNC flow was changed from 10 to 60 L/min. Capnograms were recorded at the upper pharynx, oral cavity, subglottic, and inlet sites of each lung model.

**Results:**

With the normal-lung, open-mouth model, 10 L/min of HFNC flow decreased the subglottic P_ET_CO_2_ to 30 mmHg. Increasing the HFNC flow did not further decrease the subglottic P_ET_CO_2_. With the normal-lung, closed-mouth model, HFNC flow of 40 L/min was required to decrease the P_ET_CO_2_ at all sites. Subglottic P_ET_CO_2_ reached 30 mmHg with an HFNC flow of 60 L/min. In the obstructive-lung, open-mouth model, P_ET_CO_2_ at all sites had the same trend as in the normal-lung, open-mouth model. In the restrictive-lung, open-mouth model, 20 L/min of HFNC flow decreased the subglottic P_ET_CO_2_ to 25 mmHg, and it did not decrease further. As HFNC flow was increased, PEEP up to 7 cmH_2_O was gradually generated in the open-mouth models and up to 17 cmH_2_O in the normal-lung, closed-mouth model.

**Conclusions:**

The washout effect of the HFNC was effective with relatively low flow in the open-mouth models. The closed-mouth model needed more flow to generate a washout effect. Therefore, HFNC flow should be considered based on the need for the washout effect or PEEP.

## Background

The recently invented high-flow nasal cannula (HFNC) system, which is used in respiratory therapy, is easier than noninvasive positive-pressure ventilation (NPPV) and is more effective than conventional oxygen therapies [1, 2]. The initial assumptions about HFNC for adults were that it allowed humidification and accurate F_I_O_2_ management; therefore, HFNC’s respiratory physiological effects did not become well understood. Those respiratory physiological effects are estimated to include the following: washout of the anatomical dead space of the respiratory tract, a positive end-expiratory pressure (PEEP) effect, and reduction of the metabolic cost of gas conditioning by humidification. The effect of PEEP has been evaluated by measuring airway pressure [3, 4] or by using electrical impedance tomography [5, 6]. The effects of HFNC on ventilation include a reduction of the respiratory rate [1] and minute ventilation [7] plus an improvement in thoraco-abdominal synchrony [8]. However, the mechanism has been surmised either from the study of tracheal gas insufflation (TGI) [9, 10], which is quite a different system from HFNC with “high flow” less than 8 L/min [10], or from a simple respiratory model, which had a larger dead space than the actual value in humans [11].

Thus, there is no sophisticated study that focused on the mechanism of the washout effect and was performed with an HFNC system. In clinical practice, the usage of HFNC relieves dyspnea, but the contributions of the washout effect and PEEP are unknown. Therefore, we conducted this study to evaluate the relationship between HFNC flow, the washout effect, and PEEP.

### Objective

The objective of this study is to evaluate the respiratory physiological effects of HFNC.

## Methods

Optiflow™ (Fisher and Paykel Healthcare, Auckland, NZ) was used as the HFNC system.

**Fig. 1:**
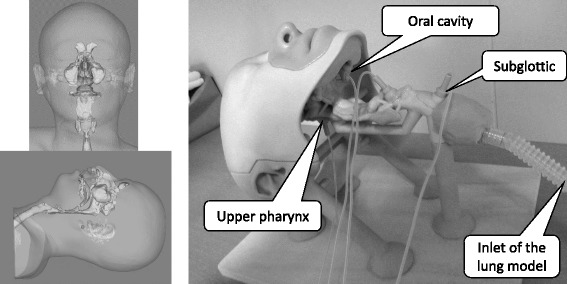
The airway model. The actual airway model was made by a 3D printer using 3D-CT data. Sampling ports were made to record capnograms at each site in the model

An airway model was made by a 3D printer (ZPrinter® 450: Z Corporation, Rock Hill, South Carolina, USA) using the craniocervical 3D-CT data of a 32-year-old, healthy male (height, 170 cm; weight, 65 kg); the volunteer was equipped with HFNC (flow, 35 L/min and F_I_O_2,_ 0.21), and CT images were obtained during the exhalation phase (Fig. 1). The materials used to make the airway model were mainly calcium sulfate hemihydrate and 2-pyrrolidone, which was solid in the finished product. The anatomical dead space was 2 mL/kg, and the physiological dead space was adjusted to 3 mL/kg. Sampling ports were inserted into the model’s upper pharynx, oral cavity, subglottic area, and inlet to measure a capnogram at each site.

**Fig. 2:**
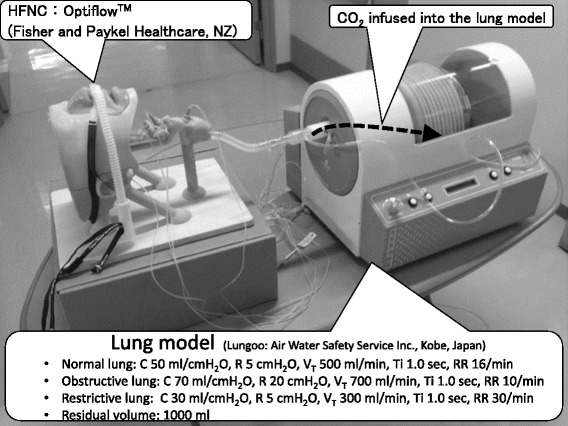
The respiratory model. Experimental system used in the study. The airway model (Fig. 1) was connected to the lung model. The physiological dead space was adjusted to 3 mL/kg. The respiratory patterns of the lung model could be changed, and the model had pressure sensors to measure the internal pressure. Sampling tubes were connected to the capnogram at each site mentioned

Lungoo (Air Water Safety Service Inc., Kobe, Japan) was used as the lung model. Lungoo consists of a bellows, a motor, and a spring. The inspiratory effort was generated by the motor, and passive exhalation was done by the spring. Resistance and compliance were controlled by the motor regulated by the controller. The lung model settings were as follows: normal (compliance [C], 50 mL/cmH_2_O; resistance [R], 5 cmH_2_O/L/s; tidal volume [Vt], 500 mL; and respiratory rate [RR], 16 breaths/min), obstructive (C, 70 mL/cmH_2_O; R, 20 cmH_2_O/L/s; Vt, 700 mL; and RR, 10/min), and restrictive (C, 30 mL/cmH_2_O; R, 5 cmH_2_O/L/s; Vt, 300 mL; and RR, 30/min). In all models, the inspiratory time (Ti) was 1.0 s and the residual volume was 1000 mL. CO_2_ was infused into the lung model to reach 40 mmHg of partial pressure of end-tidal CO_2_ (P_ET_CO_2_) measured at the upper pharynx in each model without HFNC (Fig. 2).

Two sequences (with an open and closed mouth) were performed in the normal lung model, and one sequence (with an open mouth) was performed in the obstructive and restrictive lung models. The HFNC flow was changed from 10 to 60 L/min, and the capnograms at each sampling site were measured 3 min after each HFNC flow was changed by using a bedside patient monitor (IntelliVue MP30: Royal Philips, Eindhoven, Netherlands). The generated PEEP and the pressure developed by the respiratory muscles (Pmus) that was needed to maintain the Vt were also recorded from sensors inside the lung model (Fig. 2).

## Results

### Capnograms in each lung model

**Fig. 3:**
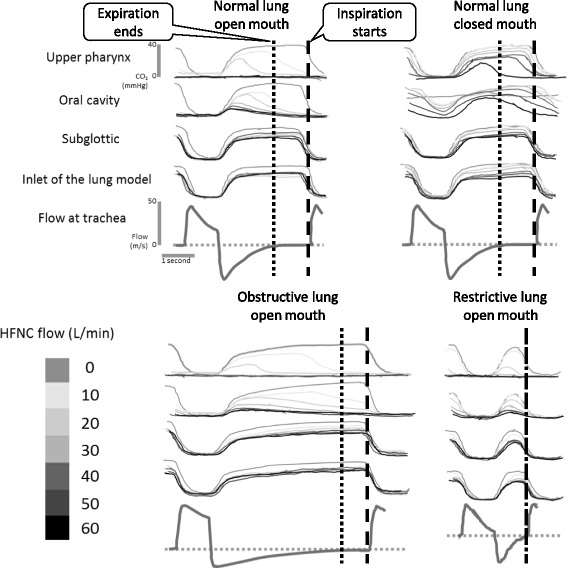
Capnograms and airway flows recorded. Capnograms were recorded with an HFNC flow of 10–60 L/min in each lung model. Airway flows were recorded without HFNC by the flow sensor in the lung model. There were no differences between the capnograms recorded at the subglottic and the inlet sites of the lung model, indicating the flow generated by HFNC does not reach further than the subglottic area. In open-mouth models, an HFNC flow of 10–20 L/min washed out the CO_2_ of the upper pharynx and the oral cavity. The closed-mouth model needed more HFNC flow to wash out the CO_2_ of the upper pharynx and the oral cavity

Capnograms in each lung model and HFNC flow are shown in Fig. 3.

#### Normal-lung, open-mouth model

Capnograms recorded at the subglottic area and the inlet of the lung model had flat phase 3 waveforms, which demonstrated that the gas delivered by HFNC does not reach and wash out the dead space below the subglottic area. With capnograms recorded at the upper pharynx and oral cavity, PCO_2_ started to decrease before phase 3 ends. An HFNC flow of 10 L/min was able to totally wash out CO_2_ by the end of phase 3 at these upper two sampling ports.

Based on these results, an HFNC flow of 10 L/min was able to fully wash out CO_2_ from the areas reached by the gas delivered by HFNC before the next inspiration started. Therefore, with the normal-lung, mouth-open condition, an HFNC setting with flow of 10 L/min was adequate to reach the maximum washout effect.

#### Normal-lung, closed-mouth model

Capnograms recorded at the subglottic area and at the inlet of the normal-lung, closed-mouth model had the same pattern as the open-mouth model, which also demonstrated that the gas delivered by HFNC does not reach and wash out the dead space below the subglottic area. Capnograms recorded at the upper pharynx and oral cavity of the normal-lung, closed-mouth model show that PCO_2_ starts to decrease before phase 3 ends, which differed from the open-mouth model in which PCO_2_ at the end of phase 3 did not reach zero until HFNC flow was raised to 40 L/min at the upper pharynx and 50 L/min in the oral cavity.

These results show that HFNC flow of 10 to 50 L/min was not able to fully wash out the CO_2_ in areas reached by the gas delivered by HFNC before the next inspiration started. Therefore, with the normal-lung, closed-mouth model as opposed to the open-mouth model, an HFNC flow of more than 50 L/min was needed to reach a maximum washout effect.

#### Obstructive-lung, open-mouth model

Capnograms recorded with an obstructive-lung, open-mouth model showed the same patterns as the normal-lung, open-mouth model at each sampling site.

#### Restrictive-lung, open-mouth model

The restrictive-lung, open-mouth model had a short phase 3; therefore, this model had less time for CO_2_ to be washed out. Contrary to other open-mouth models, this model had PCO_2_ remaining at the upper pharynx by the end of phase 3 with an HFNC flow of 10 L/min.

### Efficiency of the washout effect

Estimated from capnograms recorded at the subglottic site and the inlet of the lung model, the flow generated from HFNC did not reach below the subglottic area. Therefore, the efficiency of the washout effect can be estimated from the reduction of P_ET_CO_2_ measured at the subglottic site.

**Fig. 4:**
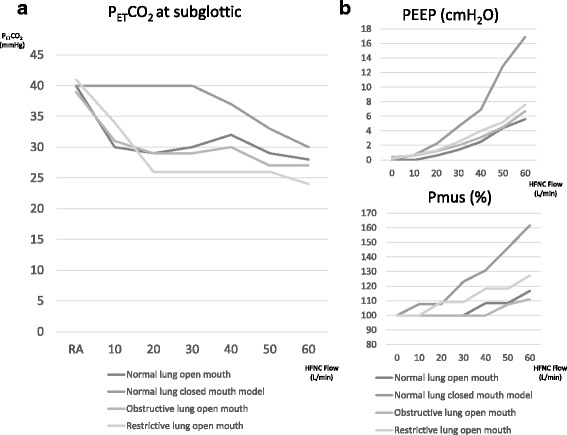
Relationship between P_ET_CO_2_, PEEP, and inspiratory effort to maintain Vt. **a** P_ET_CO_2_ measured at the subglottic site in each respiratory model. P_ET_CO_2_ in the open-mouth models reaches a minimum value with a relatively low flow. The closed-mouth model required more flow to establish the washout effect. **b** The Pmus needed to maintain the initial Vt without HFNC was counted as 100%. As HFNC flow was raised, the generated PEEP and Pmus required to maintain the initial value increased

The normal-lung, open-mouth model and the obstructive-lung, open-mouth model showed the same pattern and also had the same trend, reaching 30 mmHg with an HFNC flow of 10 L/min. With the normal-lung, closed-mouth model, P_ET_CO_2_ began to decrease with an HFNC flow of 40 L/min, but 60 L/min of HFNC flow was required to reach a P_ET_CO_2_ of 30 mmHg. With the restrictive-lung, open-mouth model, P_ET_CO_2_ continued to decrease to 26 mmHg with a higher HFNC flow of 20 L/min (Fig. 4a).

### PEEP and Pmus

As the HFNC flow increased, PEEP up to 7 cmH_2_O was generated with each open-mouth model and up to 17 cmH_2_O with the normal-lung, closed-mouth model. The Pmus that was required to maintain a Vt of 500 mL with a normal lung, a Vt of 700 mL with an obstructive lung, and a Vt of 300 mL with a restrictive lung increased as the HFNC flow was raised. The normal-lung, closed-mouth model required Pmus up to 160% of the initial value, and other models required Pmus up to around 110 to 130% (Fig. 4b).

## Discussion

This is the first study that focused on HFNC’s washout effect using a sophisticated respiratory model. In summary, the washout effect does not need a higher flow of HFNC, but PEEP requires a higher flow; however, a higher flow may increase the work of breathing (WOB). Therefore, from our study, we may say that optimal flow for each patient may differ according to the need for ventilation or oxygenation support, which was also purported by a previous clinical study [12].

The improvement of oxygenation by HFNC has been studied clinically by airway pressure and electrical impedance tomography, and all studies concluded that HFNCs are able to generate PEEP [4–6]. Prior studies found that a higher HFNC flow generates a higher PEEP, which is compatible with the results of our study (Fig. 4b) [3, 12]. On the other hand, the effect of the HFNC on ventilation has been detected by some precise clinical studies [7, 12] and a bench study with a simple respiratory model [11]; however, the mechanism has not been well described. Therefore, we now do not know how HFNC contributes to ventilation. Also, a study addressing the failure of HFNC and increased mortality has been recently published [13]. The reasons that a failure of HFNC increases mortality are still unknown: this may be caused by HFNC giving comfort and relief of dyspnea without improving the lung condition or by masking the signs of deterioration of the lung itself or even the patient’s general condition, which may mislead clinical decision-making. A study of the respiratory physiological effect of HFNC needs to be done to determine the indication for and settings of the HFNC.

Our study found that the washout effect of HFNC is effective with a relatively low flow of 10 L/min except in the normal-lung, closed-mouth model and the restrictive-lung, open-mouth model. These results were compatible with the clinical data from a previous publication that stated “most of the reduction in the effort and work of breathing can be obtained at even the lowest flow rate of 30 L/min” [12].

An HFNC flow of 10 L/min delivers 166 mL/s of fresh gas and washes out the anatomical dead space of 130 mL in only 0.78 s. In normal- and obstructive-lung, open-mouth models, there was enough time after exhalation and before the beginning of the next inspiration (Fig. 4) to reach the maximum washout effect with an HFNC flow of 10 L/min. With the restrictive-lung, open-mouth model, inspiration started right after exhalation ended; therefore, HFNC of 10 L/min was not enough, and 20 L/min was required to totally wash out the anatomical dead space and reach the maximum washout effect in this condition. A high respiratory rate and a shorter time after exhalation make the HFNC washout effect less efficient: a higher HFNC flow is required to reach the maximum effect. The normal-lung, closed-mouth model requires more HFNC flow to reduce P_ET_CO_2_ than open-mouth models. This phenomenon was thought to be because delivered gas from the HFNC must enter and exit from only the nostrils with the closed-mouth condition; thus, the closed-mouth condition is much less efficient than the open-mouth condition in which delivered gas enters through the nostrils and exits from the oral cavity. In a past study recording capnograms with TGI, intubated and mechanically ventilated patients showed a decrease of PCO_2_ during phase 3, and this reduction was said to be associated with the efficiency of reducing dead space ventilation [9, 14]. In our study, capnograms recorded at the upper pharynx and oral cavity showed the same PCO_2_ decrease at phase 3 but not at the trachea, which meant that HFNC-delivered gas was not able to reach down to the trachea. This would be a difference between HFNC and TGI, in which the gas delivered in the trachea causes turbulence for the alveolar dead space effect.

The restrictive-lung model, which had a smaller Vt, tended to have a greater drop in P_ET_CO_2_ with HFNC than other lung models (Fig. 4a). This was estimated to be due to the inversely proportional ratio of the dead space to the Vt. Because of this inverse proportion, the ratio of dead space ventilation progressively increases as Vt decreases; therefore, the magnitude of the washout effect increased, as our study showed.

The PEEP generated by HFNC had a linear correlation with HFNC flow, which was also compatible with previously published studies [3, 6, 12]. The level of generated PEEP differed dramatically between the open-mouth and closed-mouth models, which was opposite of the washout effect. Adding to this PEEP effect, another interesting finding from our study was that the Pmus needed to maintain the Vt increased as HFNC flow increased. This phenomenon meant that increasing the HFNC flow might increase the work of breathing inversely to the washout effect. Although we were not able to clarify the mechanism of this increase of the WOB from our results, the study done by Spence et al. showed that there is flow against inspiration even at the peak of inspiration due to turbulence in the nasal cavity [15]. This study did not show the change of inspiratory effort, but this counter flow may be one of the reasons for the increasing inspiratory effort.

### Limitations

One limitation of our study is that our model is not able to combine the effects of HFNC: the washout effect occurs with a relatively low HFNC flow, PEEP is generated as HFNC flow is increased, and the WOB is increased by a higher HFNC flow. All of these respiratory physiological effects demonstrated in our study would be combined in clinical practice. Additionally, the respiratory physiological condition would vary by patient, and each respiratory effect of HFNC would have a different impact. Therefore, ideal HFNC flow may differ by etiology in each patient based on the need for reduction of the WOB or PEEP.

Another limitation of our study is that our model is made from solid materials, and it was not able to replicate the change of resistance caused by airway pressure, as would occur in actual patients. Also, the lung model was not able to replicate the change in airway resistance and compliance caused by generated airway pressure. Although we made our airway model with a CT image scanned at the exhalation phase with an HFNC flow of 35 L/min to replicate an airway expanded by HFNC, the resistance of the airway is thought to be different during inspiration, exhalation, and HFNC flow. Adding to these limitations, anatomical traits vary widely in patients, affecting the impact of each respiratory physiological effect. Although it was done in a pediatric model, a study by Sivieri et al. found that the size of the nares and prong affected the PEEP generated [16]. Our model had the same trend but higher PEEP than other clinical studies done previously [3, 4]. This phenomenon may have been caused by the anatomical traits of our volunteer.

To resolve these limitations, further clinical studies are needed to investigate the different effects of HFNC flow in different patient conditions.

## Conclusions

HFNC’s washout effect occurs with a relatively low HFNC flow in contrast to PEEP, and it was also influenced by the exhalation time and Vt. Raising the HFNC flow might increase the WOB; therefore, a clinical investigation is required to determine how these respiratory physiological effects of HFNC combine in humans.

## Abbreviations

C: Compliance
HFNC: High-flow nasal cannula
NPPV: Noninvasive positive-pressure ventilation
PEEP: Positive end-expiratory pressure
P_ET_CO_2_: Partial pressure of end-tidal CO_2_
Pmus: Pressure developed by the respiratory muscles
R: Resistance
RR: Respiratory rate
TGI: Tracheal gas insufflation
Vt: Tidal volume
WOB: Work of breathing
